# Retraction: Susceptibility to Infection and Immune Response in Insular and Continental Populations of Egyptian Vulture: Implications for Conservation

**DOI:** 10.1371/annotation/d3fa7bf5-5044-4e03-bbef-0d82382172d2

**Published:** 2012-10-01

**Authors:** Laura Gangoso, Juan M. Grande, Jesús A. Lemus, Guillermo Blanco, Javier Grande, José A. Donáar

The authors wish to retract the article “Susceptibility to Infection and Immune Response in Insular and Continental Populations of Egyptian Vulture: Implications for Conservation”.

The Ethics Committee of the Spanish Superior Council of Scientific Research (CSIC) has carried out a formal investigation in relation to concerns about potential scientific misconduct by Dr. Lemus, a co-author of this article. 

Although the field and statistical procedures, as well as the immune assays, reported in the article are correct, the investigation carried out by the Ethics Committee of CSIC has not been able to clarify in which external laboratories Dr. Lemus conducted the microbiological isolation, which raises concerns regarding the validity of the analyses contributed by Dr. Lemus. During the institutional investigation, concerns were also raised about the existence of co-author Javier Grande.

Due to the ephemeral nature of the material used (bacterial microflora isolated from the cloaca, choana, and nares using microbiological swabs and Amies transport medium), we are unable to repeat the analyses with the same samples employed in the original study. As a result, the authors wish to retract this article.

